# Molecular and phylogenetic characterization of *Thelohanellus qadrii *(Myxozoa, Myxosporea, Bivalvulida) infecting the secondary gill epithelium of Indian major carp, *Catla catla *(Hamilton, 1822)

**Published:** 2015-06

**Authors:** Sayani Banerjee, Avijit Patra, Harresh Adikesavalu, Siddhartha Narayan Joardar, Thangapalam Jawahar Abraham

**Affiliations:** Department of Aquatic Animal Health, Faculty of Fishery Sciences, West BengalUniversity of Animal and Fishery Sciences, Chakgaria, Kolkata, India

**Keywords:** *Catla catla*, Myxosporean infection, *Thelohanellus qadrii*, Molecular characterization, Phylogenetic relationship

## Abstract

Myxosporean taxonomy which is traditionally based on the morphology of the myxospore stage, is in a state of flux given new insights provided by the expanding dataset of DNA sequences. To date, more than 40 species of *Thelohanellus *from India have been described according to morphometric characteristics. Nevertheless, molecular data on these histozoic myxosporean parasites of freshwater fish are scarce. In the present study, molecular characterizations of *Thelohanellus qadrii *infecting the secondary gill epithelium of Indian major carp *Catla catla *(Hamilton, 1822) and its phylogenetic relationship is reported. The sub-adult cultured catla were observed to have low to moderate gill myxosporean infections. The morphometry of mature spores was in compliance with original descriptions of *T. qadrii. *Based on the analysis of 18S rRNA gene, phylogenetic clusters which were established according to a consensus sequence, illustrated the taxonomic placement of a series of myxobolids. The DNA sequence homogeneity of *T. qadrii *(KF170928) with other *Thelohanllus *spp. ranged from 78% to 95% and formed a dichotomy with cyprinid gill lamellae infecting *T. toyamai *(HQ338729). Distance matrix results indicated a high genetic diversity among myxosporeans. The present report is the first on the molecular and phylogenetic characterizations of *T. qadrii*.

## INTRODUCTION

Over 62 genera of phylum Myxozoa have been described from fishes, primarily on the basis of myxosporean spore structure [1]. Among them, species of *Thelohanellus *Kudo, 1933 are typically histozotic (rarely coelozoic) bivalvulidan myxosporean parasites of freshwater fish with a high diversity of infection sites [2]. In recent years, myxosporeans have received much attention as emerging fish pathogens as they are continually threatening the development of aquaculture [3-6]. The increased importance of myxosporean diseases has initiated scientific research on life cycles, host-pathogen relationships and the development of diagnostic tests for myxosporean parasites. Molecular systematics using small subunit ribosomal DNA sequences and sensitive PCR tests are currently used to resolve specific relationships within the Myxozoa and to identify the parasites [3, 7-10]. *Thelohanellus *is the sixth most speciose myxozoan genus with 108 nominal species reported worldwide to date, of which 40 species are from India [11]. Molecular studies on Indian myxosporeans are rare. The authors have recently reported on the molecular characterization of *T. caudatus *infecting the caudal fin of *Labeo rohita *[12]. In this article, for the first time we present the molecular characterizations of *Thelohanellus qadrii *infecting the secondary gill epithelium of Indian major carp *Catla catla *(Hamilton, 1822) and its phylogenetic relationship.

## MATERIALS AND METHODS

A total of 60 juvenile to sub-adult carp (*Catla catla*) from a polyculture pond in Garia (Lat. 22°27’59’’N; Long. 88°24’18’’E), Kolkata, West Bengal, India, were screened during the routine survey of carp myxosporean infections in 2013. Myxosporeans infecting the secondary gill epithelia of catla were collected and characterized by morphometric techniques. Myxosporean identification was performed according to Lom & Arthur [13]. In brief, a fresh plasmodium was first taken on a clean grease-free glass slide with a few drops of distilled water and then slightly ruptured. The spores released from the plasmodium were then spread onto clean grease free glass slides, covered with cover slips and sealed with Distrene, Plasticizer and Xylene (DPX) for examination under an oil immersion (100X) lens. Two fresh spore smears were treated with 2% KOH (w/v) for polar filament extrusion. The Indian ink method was employed to observe the mucous membrane around the spores. Smears of fresh spores were treated with Lugol’s iodine solution to observe iodinophilic vacuoles in the sporoplasm. For permanent slides, air dried smears were fixed with acetone free absolute methanol for about 8 min and stained with Giemsa solution for 40 min afterwards. The Giemsa solution was prepared by dissolving 0.5 g Giemsa powder in 33 mL glycerol at 50-60ºC for 90 min in a water bath followed by the addition of 33 mL methanol. This solution was matured in the dark for 15 days and diluted with phosphate buffer (pH 7.2) with a 1:2 ratio prior to use. The slides containing myxosporean spores were observed under an oil immersion (100X) lens of a Motic BA400 microscope withan inbuilt digital camera. Morphometric measurements were done in μm by Motic Image Plus Version 2 software.

Morphologically identified myxosporeans was further characterized by molecular techniques as described previously [12]. After morphometric confirmation of the first plasmodium spores, the spores were suspended in a 500 μL lysis buffer (100 mM NaCl,10 mM Tris, 10 mM EDTA, 0.2% SDS, 0.4 mg/mL Proteinase K) and incubated overnight at 55°C. Afterwards, 500 μL of phenol:chloroform (1:1) was added to the digested spores, mixed gently and centrifuged at 5200g for 10 min. The upper phase was later transferred to a new tube and mixed with a 1/10 volume of sodium acetate (3 M, pH 5.2) and 2 volumes of 96% ethanol (Amresco, USA). DNA was precipitated at –20°C overnight and pelleted by centrifugation at 10000g for 30 min. The pellet was washed once with 70% ethanol, air-dried for several minutes and resuspended in 30 μL of molecular biology grade water. The universal eukaryotic primers -ERIB1, 5´-ACC TGG TTG ATC CTG CCA G-3´ and ERIB10, 5´-CTT CCG CAG GTT CAC CTA CGG-3´ [14] were used for the amplification of 18S rDNA by Eppendorf Master cycler Pro S. The PCR was run using a mixture of 50 ng of genomic DNA, 10 μM of each primer and a 2X PCR TaqMixture. Amplification was done by initial denaturation at 95°C for 5 min, followed by 35 cycles of denaturation at 95°C for 30 sec, annealing of primers at 51°C for 30 sec and extension at 72°C for 60 sec. The final extension was at 72°C for 5 min. PCR product was analysed on a 1.5% agarose gel containing 0.5 μg/mL ethidium bromide in a 1X Tris-acetate-EDTA (TAE) buffer. Following the purification of the amplified PCR product by an EXO-SAP treatment, the DNA was quantified and subjected to automated DNA sequencing on an ABI 3730xl Genetic Analyzer (Applied Biosystems, USA). BigDye® Terminator v3.1 Cycle sequencing kit (Applied Biosystems, USA) was used for sequencing as per the manufacturers’ instructions. Electrophoresis and data analyses were carried out on the ABI 3730xl Genetic Analyzer.

Phylogenetic analysis was performed on a selection of 18S rRNA sequences that comprised the new sequence (KF170928) and 17 additional sequences from closely related species available in the NCBI GenBank database using the basic local alignment search tool (BLAST) and other representatives of the Myxobolidae clade (Table 1) as described by Fiala [9]. *Buddenbrockia plumatellae *(AY074915) of the class Malacosporea was used as an out-group.

The sequences were compared by standard nucleotide BLAST (www.ncbi.nlm.gov/ BLAST/). Data analysis and multiple alignments were performed by ClustalX [15] and MEGA5 [16] software, respectively. Genetic distance analyses were conducted using the Kimura 2-parameter model [17]. Codon positions included were 1st + 2nd + 3rd + Noncoding. All positions containing gaps and missing data were eliminated. Evolutionary history was inferred using the maximum likelihood method.

A bootstrap consensus tree inferred from 1000 replicates was taken to represent the evolutionary history of the taxa analyzed. Branches corresponding to partitions reproduced in <50% bootstrap replicates were collapsed. The percentage of replicate trees in which the associated taxa were clustered together in the bootstrap test (1000 replicates) is shown next to the branches [18]. The nucleotide sequence generated in the present study was deposited in the NCBI GenBank database under accession number KF170928.

## RESULTS AND DISCUSSION

**Figure 1:**
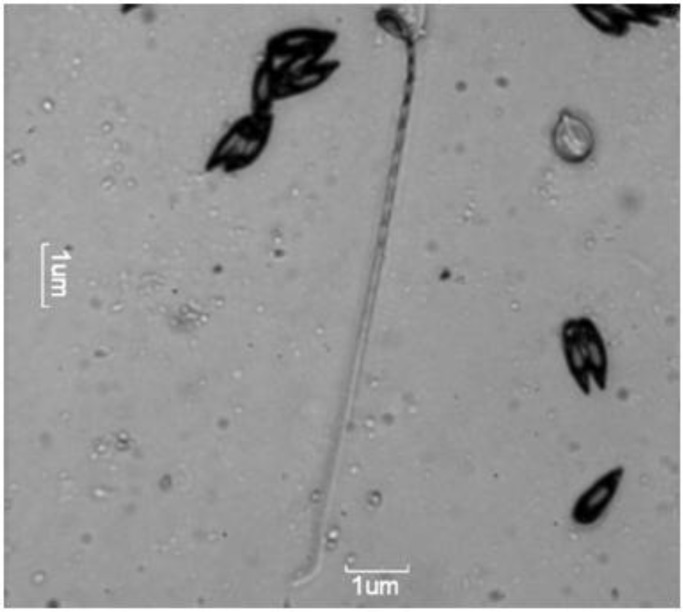
Wet mount preparation showing mature spores of *Thelohanellus* *qadrii* with extended polar filament at 200X magnification (bar = 1 µm

During the survey, 25 out of 60 cultured catla (41.66%) were observed to have low to moderate gill myxosporean infection. The plasmodia found on the secondary gill epithelia were very small (0.2-0.3 mm), whitish and oval shaped. Mature spores measured 14.80 ± 0.61 (14.0-15.9) × 5.52 ± 0.25 (5.0–5.9) μm. Spores were elongated pyriforms in valvular view with rounded posteriors and tapering anterior ends. In the sutural view, they appeared slightly lenticular with indistinct sutural lines. The polar capsule with a size of 8.51 ± 0.42 (7.6-8.9) × 3.22 ± 0.19 (3.0-3.7) μm was situated at the anterior portion and was similar to the shape of the spore. The flat anterior surface was situated at a short distance below the anterior end of the spore. The polar filament formed 14-16 coils within the capsule. The 93 μm long thread-like polar filament was wavy and tapering when completely extruded (Fig. 1). The sporoplasm contained two large nuclei and a large irregular shaped iodinophilous vacuole. Mucus envelopes around the spore and intercapsular processes (ICP) were absent. Based on morphometry, the myxosporean infecting the secondary gill epithelium was identified as *Thelohanellus qadrii.*

**Figure 2:**
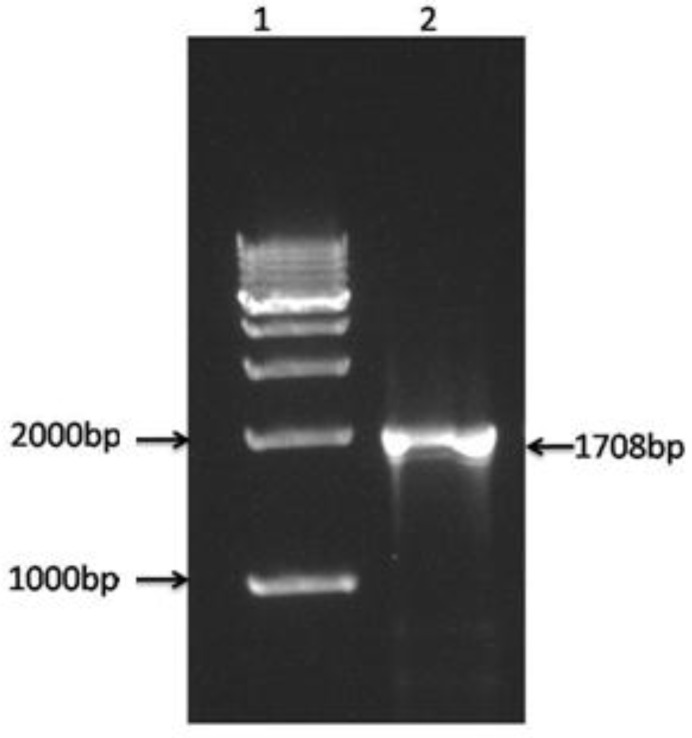
Agarose gel (1.5%) showing 18S rRNA gene amplification of *Thelohanellus* *qadrii*

**Figure 3:**
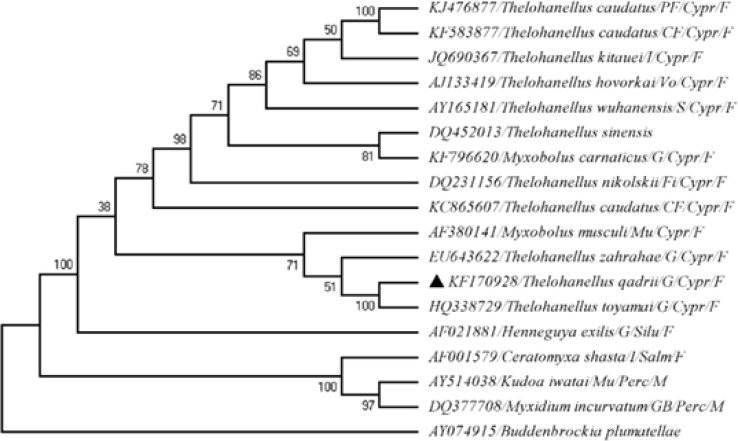
Phylogenetic tree generated by maximum likelihood of the 18S rRNA gene sequences of

Universal primers successfully amplified approximately 1708 bp fragments (Fig. 2) of the 18S rRNA gene of *T. qadrii*. The phylogenetic cluster was established based on the 1703 bp consensus sequence. The phylogenetic tree (Fig. 3) illustrated the taxonomic placement of a series of myxobolids based on the analysis of the 18S rRNA gene. The novel DNA sequence of *T. qadrii *clustered phylogenetically with other *Thelohanllus *spp. with 78-95% homogeneity (Table 1). Evolutionary pair-wise distances among *T. qadrii *and other species of myxosporeans, measured by Kimura-2 parameter algorithm (Table 1), ranged from 0.00 (*T. toyamai *HQ338729) to 0.56 (*Kudoa iwatai *AY514038).

(1708bp) from *Catla catla*. 1: DNA ladder, 2: *Thelohanellus qadrii*

Although morphological data on many Indian *Thelohanellus *spp. are available [2,5, 11], molecular data are not. Prior to molecular characterization, we morphometrically characterized the myxosporean species isolated from the secondary gill epithelium of carp. The spore length (LS) to breadth (BS) ratio (1:0.37) and the length of polar capsules (LPC) to their breadth (BPC) ratio (1:0.38) were, more or less, in conformity with the original descriptions of *Thelohanellus qadrii *(LS:BS = 1:0.36; LPC:BPC =1:0.46), a species described by Lalithakumari (1969) [19] from *Labeo potail *in Andhra Pradesh, India. Although the spore size slightly differed, it did not exceed the limits of natural variations typical of species' populations. Several other gill-infecting *Thelohanellus *spp. from Indian cyprinids have been described [11]. In the present study, a comparison of the morphometric data of *T. qadrii *with the representatives of *Thelohanellus *spp. infecting the gills of cyprinids [11] further revealed its morphometrical difference with the others. These observations, thus, confirmed that the *Thelohanellus *species found on the gills of cultured catla was *T. qadrii *in terms of morphology, host (carp) specificity and tissue (gill) tropism.

**Table 1:** Homogeneity of 18S rRNA gene sequences of selected myxozoan species available in NCBI GenBank and *Thelohanellus qadrii* (Accession number KF170928) and estimates of evolutionary divergence among the select 18S rRNA gene sequences of Myxosporea and Malacosporea available in NCBI GenBank

	Myxozoan species	A*	1	2	3	4	5	6	7	8	9	10	11	12	13	14	15	16	17	18
1	KF170928/*Thelohanellus qadrii*	100	**0.00**																	
2	KJ476877/*Thelohanellus* *caudatus*	85	**0.25**	0.00																
3	KF583877/*Thelohanellus* *caudatus*	85	**0.25**	0.00	0.00															
4	KC865607/*Thelohanellus* *caudatus*	78	**0.43**	0.13	0.13	0.00														
5	JQ690367/*Thelohanellus* *kitauei*	87	**0.22**	0.04	0.04	0.14	0.00													
6	AJ133419/*Thelohanellus* *hovorkai*	87	**0.26**	0.07	0.07	0.18	0.04	0.00												
7	HQ338729/*Thelohanellus toyamai*	95	**0.00**	0.25	0.25	0.43	0.22	0.25	0.00											
8	AY165181/*Thelohanellus wuhanensis*	84	**0.27**	0.10	0.10	0.22	0.06	0.07	0.27	0.00										
9	DQ231156/*Thelohanellus* *nikolskii*	86	**0.27**	0.12	0.12	0.27	0.10	0.13	0.26	0.14	0.00									
10	EU643622/*Thelohanellus* *zahrahae*	89	**0.20**	0.20	0.20	0.40	0.20	0.21	0.19	0.25	0.23	0.00								
11	DQ452013/*Thelohanellus* *sinensis*	87	**0.23**	0.12	0.12	0.25	0.09	0.11	0.22	0.12	0.13	0.20	0.00							
12	KF796620/*Myxobolus* *carnaticus*	88	**0.23**	0.11	0.11	0.25	0.09	0.09	0.22	0.12	0.12	0.18	0.09	0.00						
13	AF380141/*Myxobolus musculi*	87	**0.21**	0.29	0.29	0.53	0.30	0.31	0.20	0.33	0.30	0.22	0.29	0.26	0.00					
14	AF021881/*Henneguya exilis*	85	**0.26**	0.27	0.27	0.45	0.24	0.26	0.26	0.28	0.30	0.27	0.25	0.26	0.32	0.00				
15	AY514038*/Kudoa iwatai*	82	**0.56**	0.62	0.62	0.89	0.60	0.62	0.55	0.67	0.57	0.60	0.61	0.60	0.63	0.64	0.00			
16	AF001579/*Ceratomyxa shasta*	81	**0.51**	0.57	0.57	0.85	0.57	0.62	0.50	0.65	0.64	0.58	0.60	0.58	0.55	0.63	0.35	0.00		
17	DQ377708/*Myxidium incurvatum*	81	**0.53**	0.55	0.55	0.77	0.52	0.53	0.52	0.57	0.51	0.57	0.52	0.53	0.59	0.53	0.28	0.37	0.00	
18	AY074915/*Buddenbrockia plumatellae*	79	**0.65**	0.73	0.73	1.00	0.71	0.76	0.65	0.73	0.78	0.72	0.72	0.71	0.65	0.70	0.80	0.73	0.64	0.00

A* : DNA sequence homogeneity (%) to *Thelohanellus qadrii *(KF170928).

This is the first report regarding the molecular and phylogenetic characterization of *T. qadrii*. Earlier, we characterized *T. caudatus *(KC865607) infecting the caudal fin of *L. rohita *[12]. In this study, approximately 1708 bp fragments of the 18S rRNA gene of *T. qadrii *were successfully amplified. Our phylogenetic tree, established by the maximum likelihood method, defined topologies resembling those generated by Fiala [9]. According to Eszterbauer [8], site specificity is an important factor in myxozoan speciation. The evolutionary tree of this study further demonstrated that tissue tropism plays an important role in genetic relationships among myxozoan species. *Thelohanellus qadrii *clustered with cyprinid gill infecting myxosporeans with 89-95% homogeneity. The phylogenetic tree placed *T. qadrii *within the freshwater clade. It formed a dichotomy with the closely related *T. toyamai *(HQ338729) that infected the gill lamellae of common carp, *Cyprinus carpio *from the USA with maximum node support. In addition, *T. qadrii *exhibited maximum DNA sequence homogeneity (95%) with *T. toyamai *(HQ338729). Our previously characterized fin infecting *T. caudatus *(KC865607) exhibited 78% homogeneity with *T. qadrii*. Furthermore, freshwater and marine clades (*Myxidium incurvatum *(DQ377708) and *Kudoa iwatai *(AY514038)) were distinctly separated within the lineage Myxosporea. Other representatives of the Myxobolidae clade such as *Ceratomyxa, Henneguya, Kudoa, Myxidium *and *Myxobolus *were also distinctly different from the gill-infecting *Thelohanellus *spp. and clustered separately. The out-group *B. plumatellae *(AY074915) of the Malacosporea class was distinctly and phylogenetically clustered as a separate lineage with Myxosporea. The wide range observed in the evolutionary pair-wise distances between *T. qadrii *and other myxosporeans (0.00-0.56) including the gill infecting *Myxobolus carnaticus *(0.23) and the fin infecting *T. caudatus *(0.25-0.43) from India possibly indicated the high genetic diversity of myxosporeans.

Myxosporeans are best known for the diseases they cause in commercially important fish hosts. With the huge global expansion of freshwater aquaculture, several myxosporeans have been recognized or elevated in status as important pathogens [5, 6]. Since the gills of catla had low to moderate infections (with the parasitic frequency

assumed. Although no mortality was noticed, such infection may cause substantial production loss. Further studies are warranted to establish this pathogenicity and its role in production loss.

## Notes

**Acknowledgements: **The work was supported by the Indian Council of Agricultural Research, Government of India, New Delhi, under the Niche Area of Excellence programme. The authors thank the Vice-Chancellor, West Bengal University of Animal and Fishery Sciences, Kolkata, for providing necessary infrastructure facility to carry out the work.

## References

[1] Lom J, Dyková I (2006). Myxozoan genera: definition and notes on taxonomy, life-cycle terminology, and pathogenic species. Folia Parasitol.

[2] Basu S, Modak BK, Haldar DP (2006). Synopsis of the Indian species of the genus Thelohanellus Kudo,1933 along with the description of Thelohenellus disporomorphus sp. n. J Parasitol Appl Anim Biol.

[3] Molnár K, Marton S, Székely C, Eszterbauer E (2010). Differentiation of Myxobolus spp. (Myxozoa: Myxobolidae) infecting roach (Rutilus rutilus) in Hungary. Parasitol Res.

[4] Liu Y, Whipps CM, Liu WS, Zeng LB, Gu ZM (2011). Supplemental diagnosis of a myxozoan parasite from common carp Cyprinus carpio: Synonymy of Thelohanellus xinyangensis with Thelohanellus kitauei. Vet Parasitol.

[5] Singh R, Kaur H (2012). Thelohanellus (Myxozoa: Myxosporea: Bivalvulida) infections in major carp fish from Punjab wetlands (India). Protistol.

[6] Yokoyama H, Grabner D, Shirakashi S, Carvalho ED, David GS, Silva RJ (2012). Transmission Biology of the Myxozoa. Health and Environment in Aquaculture.

[7] Kent ML, Andree KB, Bartholomew JL, El-Matbouli M, Desser SS, Devlin RH, Feist SW, Hedrick RP, Hoffmann RW, Khattra J, Hallett SL, Lester RJG, Longshaw M, Palenzeula O, Siddall ME, Xiao C (2001). Recent advances in our knowledge of the Myxozoa. J Eukary Microbiol.

[8] Eszterbauer E (2004). Genetic relationship among gill-infecting Myxobolus species (Myxosporea) of cyprinids: molecular evidence of importance of tissue-specificity. Dis Aquat Org.

[9] Fiala I (2006). The phylogeny of Myxosporea (Myxozoa) based on small subunit ribosomal RNA gene analysis. Int J Parasitol.

[10] Cech G, Molnár K, Székely C (2012). Molecular genetic studies on morphologically indistinguishable Myxobolus spp infecting cyprinid fishes, with the description of three new species, M alvarezae sp nov, M sitjae sp nov and M eirasianus sp nov. Acta Parasitol.

[11] Zhang JY, Gu ZM, Kalavati C, Eiras JC, Liu Y, Guo QY, Molnár K (2013). Synopsis of the species of Thelohanellus Kudo, 1933 (Myxozoa: Myxosporea: Bivalvulida). Sys Parasitol.

[12] Mondal A, Banerjee S, Patra A, Adikesavalu H, Ramudu KR, Dash G, Joardar SN, Abraham TJ (2014). Molecular and morphometric characterization of Thelohanellus caudatus (Myxosporea: Myxobolidae) infecting the caudal fin of Labeo rohita (Hamilton). Protistol.

[13] Lom J, Arthur JR (1989). A guideline for the preparation of species descriptions in Myxosporea. J Fish Dis.

[14] Barta JR, Martin DS, Liberator PA, Dashkevicz M, Anderson JW, Feighner SD, Elbrecht A, Perkins-Barrow A, Jenkins MC, Danforth HD, Ruff MD, Profous- Juchelka H (1997). Phylogenetic relationships among eight Eimeria species infecting domestic fowl inferred using complete small subunit ribosomal DNA sequences. J Parasitol.

[15] Thompson JD, Gibson JJ, Plewniak F, Jeanmougin F, Higgins DG (1997). The ClustalX windows interface: flexible strategies for multiple sequence alignment aided by quality analysis tools. Nucleic Acids Res.

[16] Tamura K, Peterson D, Peterson N, Stecher G, Nei M, Kumar S (2011). MEGA5: Molecular evolutionary genetics analysis using maximum likelihood, evolutionary distance, and maximum parsimony methods. Mol Biol Evol.

[17] Kimura M (1980). A Simple method for estimating evolutionary rate of base substitutions through comparative studies of nucleotide sequences. J Mol Evol.

[18] Felsenstein J (1985). Phylogenies and the method. Am Nat.

[19] Lalithakumari PS (1969). Studies on parasitic protozoa (Myxosporidia) of freshwater fishes of Andhra Pradesh, India. Rivista di Parasitol.

